# Supplementary material for: Causes and consequences of sex-chromosome turnovers in Diptera
Source: Evol Lett. 2026 Mar 12;10(3):253–68. doi: 10.1093/evlett/qrag003 (PMC13231274; doi:10.1093/evlett/qrag003)
Supplement: qrag003_Supplemental_Files [file qrag003_supplemental_files.zip › Supplementary_Datasets.zip!/Supplementary_Datasets/Dataset_2_Graphical_representation_outgroups/Syrphidae_Cheilosia_pagana_chromosomes.pdf]

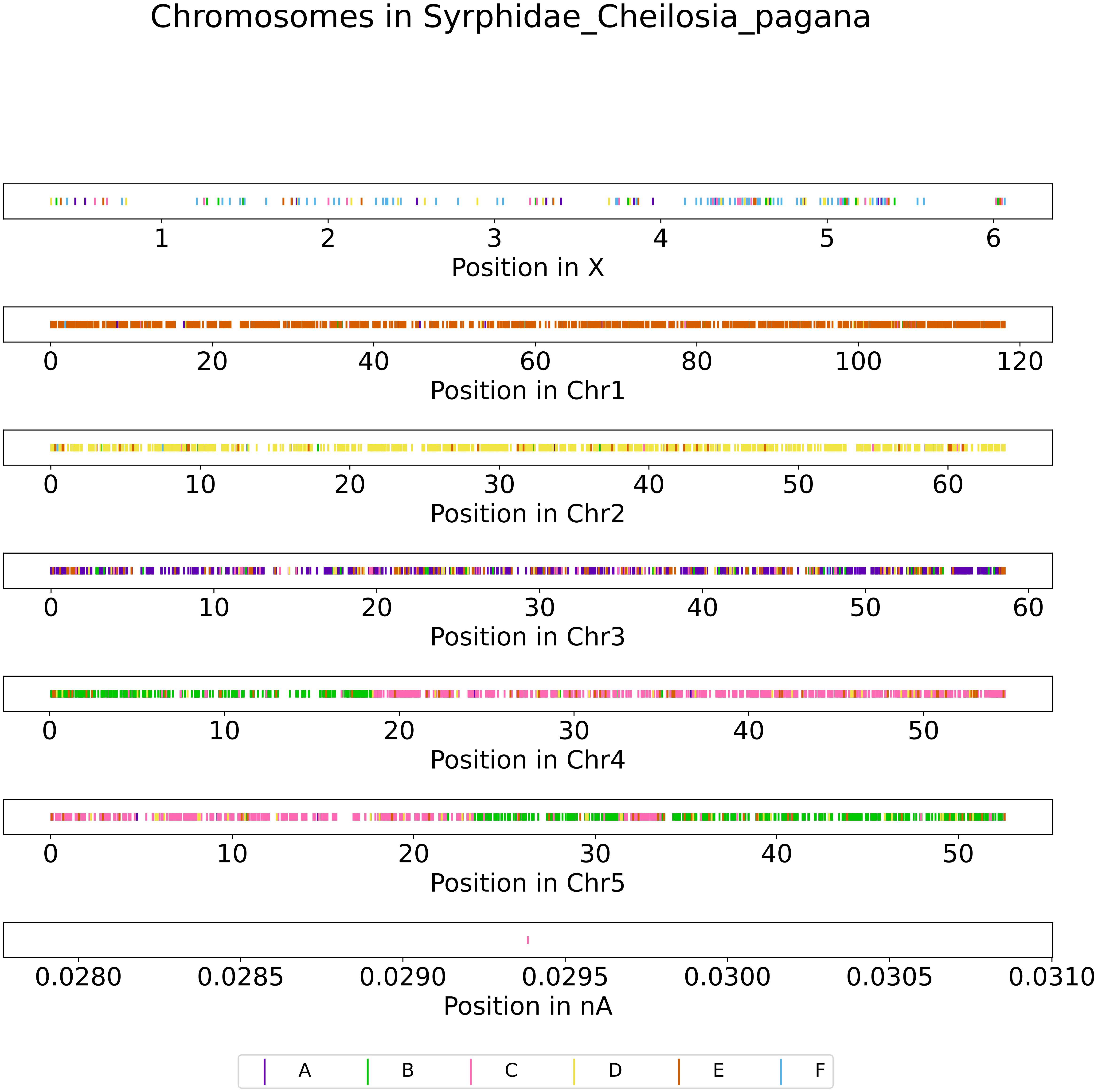
Chromosomes in Syrphidae_Cheilosia_pagana
1
2
3
4
5
6
Position in X
0
20
40
60
80
100
120
Position in Chr1
0
10
20
30
40
50
60
Position in Chr2
0
10
20
30
40
50
60
Position in Chr3
0
10
20
30
40
50
Position in Chr4
0
10
20
30
40
50
Position in Chr5
0.0280
0.0285
0.0290
0.0295
0.0300
0.0305
0.0310
Position in nA
A
B
C
D
E
F
